# Bacterial contamination of lightproof covers for high-calorie infusion solutions in wards

**DOI:** 10.7150/ijms.62193

**Published:** 2021-09-23

**Authors:** Kengo Hosomi, Yuko Takasu, Yumiko Hisai, Sachiko Komaki, Hiroaki Otsuki, Kyoko Okimoto, Sachiko Omotani, Yasutoshi Hatsuda, Michiaki Myotoku

**Affiliations:** 1 Department of Pharmacy, Kawanishi City Hospital.; 2 Faculty of Pharmacy, Osaka Ohtani University.; 3 Department of Pharmacy, Takarazuka City Hospital.; 4 Department of Clinical Laboratory, Kawanishi City Hospital.

**Keywords:** lightproof covers, high-calorie infusion solutions, bacterial contamination, *Bacillus species*, * Coagulase-negative staphylococci*, Methicillin-resistant *Staphylococcus aureus*

## Abstract

Deterioration of drugs due to light exposure is one of the major concerns, especially regarding protection of high-calorie infusion solutions, lightproof covers are used in hospitals. In the absence of any set standards regarding their usage, they are often reused. This study aimed to investigate bacterial contamination of lightproof covers used in hospital wards. For this, lightproof covers which had been used or stored in wards were collected and bacterial cultures were carried out from them. Examination of the cultures revealed that bacteria were present in the used lightproof covers. The bacterial species detected in the used lightproof covers were *Bacillus* species Coagulase-negative *Staphylococci* (CNS) and Methicillin-resistant *Staphylococcus aureus* (MRSA). *Bacillus* species and CNS were also detected in lightproof covers stored in wards, whereas MRSA was not detected. Intestinal bacteria were detected in only one lightproof cover. However, no bacteria were detected from either inside or outside of the unused lightproof covers that were stored in the drugs department. After allowing the unused lightproof covers stored in the drugs department to stand for 24 h, *Bacillus* species and CNS were detected in only one of the covers, whereas no bacteria was detected in other covers. These results indicate that there is a risk of bacterial contamination in the reuse of lightproof covers and that they should either be disposed off properly after usage or hand, finger disinfectants should be used while handling them to prevent any possible contamination.

## Introduction

Pharmacists' roles in hospitals include ensuring medical safety, and their involvement in nosocomial infection control measures is of particular importance. Some pharmaceutical ingredients are susceptible to light, and lightproof covers are an effective means to prevent light-induced alterations 1-3. However, no clear standards have been established for lightproof covers used for infusion solutions. Lightproof covers are used to cover infusion preparations requiring protection from light, such as infusion preparations mixed with multiple vitamin preparations, but reuse is possible due to their nature and so the risk of bacterial contamination caused by reuse should be considered. However, it was clarified that lightproof covers are handled differently by ward and the handling method varies among nurses. Moreover, infusion preparations using lightproof covers are placed mainly at the bedside of individual patients in hospital rooms, so that patients themselves and their families have many opportunities to contact them, in addition to healthcare workers, such as nurses and physicians. Furthermore, healthcare workers may contact the cover during exchanging transfusion or additional administration through a side tube, creating an environment easily contaminated by bacteria. However, there are no reports studying bacterial contamination of lightproof covers during and after use.

In this study, to clarify the criteria for proper use of lightproof covers, we conducted a study on bacterial contamination of lightproof covers collected after use or stored without use in the wards of Kawanishi City Hospital. Following this, bacterial culture tests were performed on the collected covers.

## Methods

### Lightproof covers studied

In total, 23 lightproof covers collected from 4 wards were included in the bacterial contamination study: 9 lightproof covers used in 2 wards (SU (U = used) covers), 8 used lightproof covers stored in 4 wards (SK (K = keep) covers), and control groups comprising 3 unused lightproof covers stored in the pharmacy department (CP (P = Pharmacy) covers) and 3 unused lightproof covers stored in the pharmacy department and placed in a ward for 24 hours (CW (W = ward) covers). A lightproof cover applied for high-calorie infusion preparation, FULCALIQ^®^ (Terumo Co., Ltd., Tokyo, Japan), was used. Lightproof covers were primarily made from polyethylene terephthalate and polyethylene. The temperature of the lightproof cover storage place was set at room temperature on both the ward and in the pharmacy department. The lightproof covers were stored in a drawer exclusive for them on the ward and in exclusive corrugated cardboard boxes with protection from light in the pharmacy department. The number of uses was unknown for the lightproof covers used or stored in the wards. The CW covers were installed in the hospital rooms by hanging only the lightproof covers on the infusion stands in the hospital rooms.

### Collection and bacterial culture testing methods

The swab method 4 was used for collecting bacteria. After collecting the lightproof covers from the ward and pharmacy department, the following procedure was performed at a clean bench in a bacteriological examination room. The outside of the lightproof covers was evenly rubbed with sterilized gauze moistened with sterilized purified water for sampling. Then, the cover was cut open with sterilized scissors and the same procedure was applied to the inside.

The swab samples were subjected to enrichment culture under aerobic conditions at 36 °C for 18 hours, followed by isolation culture at 36 °C for 24 hours. Cell colonies were stained using Gram stain, and were observed microscopically to identify bacterial species based on the staining pattens and morphology. Brain heart infusion broth for general bacteria (Nippon Becton Dickinson Co., Ltd., Tokyo) was used as the enrichment culture medium, and sheep blood agar (Nissui Pharmaceutical Co., Ltd., Tokyo) and MacConkey agar (Nippon Becton Dickinson Co., Ltd., Tokyo) were used as the isolation culture media. MS-CFX agar (Nissui Pharmaceutical Co., Ltd., Tokyo) was used for isolating methicillin-resistant *Staphylococcus aureus* (MRSA).

## Results

The bacterial culture results for SU covers (1-9) are shown in Table 1. Bacteria were detected from the outer surface of all SU covers. Bacterial species detected included *Bacillus* species, coagulase-negative staphylococci (CNS), and MRSA; these bacteria were detected from the outer surface of 9, 4, and 7 covers, respectively, and from the inner surface of 7, 6, and 4 covers, respectively, out of the 9 lightproof covers. The same bacterial species were detected from the same used covers' outer and inner surfaces, with the exception of 4 covers, in which different bacteria were detected. The usage duration (days) of the lightproof covers was unknown.

The bacterial culture results for SK covers (1-8) are shown in Table 2. Within the same covers, the same *Bacillus* species and CNS were detected on the inner and outer surface, with the exception of MRSA, and the growth of enteric bacteria was observed in one cover.

The bacterial culture results for CP covers (1-3) and CW covers (1-3) are shown in Table 3. No bacteria were detected from the inner and outer surfaces of the CP covers. *Bacillus* species and CNS were detected from the outer surface of only one CW cover, and no bacteria were detected from the inner and outer surfaces of the other CW covers.

## Discussion

Lightproof covers are essential for securing the efficacy of drugs. Particularly, the use of lightproof covers effectively prevents decomposition of multivitamins added to frequently used high-calorie infusion solutions (FULCALIQ^®^ infusion solution pharmaceutical interview form (the fourth edition), Terumo, revised in April 2019) 5.

In this survey, the adhesion of bacterial species, such as *Bacillus species*, CNS, and MRSA, to the lightproof covers was frequently detected. Various studies on the occurrence of nosocomial infection due to these species have been reported 6-9. In some cases, different bacteria were found on the same lightproof cover's inner and outer surfaces, suggesting that bacteria were attached to the respective surfaces in different conditions. More specifically, bacterial contamination of the outer surface was primarily mediated by the hands of patients and healthcare professionals, such as nurses. A previous study has reported that MRSA was isolated from 42% of the gloves worn by nurses who only had contact with the surrounding environment and no contact with MRSA carriers 10. Therefore, healthcare professionals should thoroughly wash their hands before preparing infusion solutions. Additionally, infusion solution containers on the patient's bedside should be disinfected after being touched by healthcare professionals. Measures undertaken with the assumption of bacteria being present on lightproof covers could prevent the spread of infection.

Furthermore, the inner surface of lightproof covers may be contaminated with bacteria during the preparation of infusion mixtures. At our hospital, mixtures of high-calorie infusion solutions are prepared by nurses in the wards. Hence, bacteria are likely to get attached to the infusion container's surface during the preparation of infusion mixtures and then spread to the inner surface of the lightproof cover when the infusion container is covered by the lightproof cover. However, it is challenging to prevent bacterial contamination during the preparation of infusion mixtures in wards. Hence, the entire operating procedures for the preparation of infusion mixtures in wards should be reviewed, including the adequacy of disinfection steps, such as those used for infusion solution containers, gloves, and workbenches. Based on the contamination status of the lightproof covers in this study, the method of handling was discussed by the infection control committee of our hospital, and it was decided to use disposal gloves for handling infusion preparations, such as mixing formulation, and thorough hand hygiene was promoted. Also, high-calorie infusion solutions are to be prepared in the pharmacy department and then delivered to the ward. Furthermore, used lightproof covers cannot be returned to the shelf for spares. Following strict implementation of these measures, the use of lightproof covers without detectable bacteria, similar to the ones stored in the pharmacy department, would be possible, and the risk of bacterial contamination would be minimized.

The bacterial strains adhering to the lightproof covers were identified, but the bacteria were not quantitated because of the small number of samples. We did not examine the infection status of patients for whom we used the lightproof covers, nor perform antibiotic susceptibility tests for bacterial species that were detected. As MRSA and CNS were not detected in the direct culture (data not shown), these bacteria are likely to be present in small numbers. However, they still pose a risk for infection, which cannot be overlooked. Although bacteria were detected regardless of the usage duration, the risk of spreading bacterial contamination to the environment likely increases with a lightproof cover being reused for a longer duration 11-12. In particular, *Bacillus* species and CNS, frequently detected in this study, are known to be resident bacteria; however, they have also been reported to cause catheter infection 13-14, suggesting that contaminated lightproof covers can mediate nosocomial infections. Therefore, hands and fingers should be thoroughly disinfected, particularly before infusion fluid exchange. Intestinal bacteria were detected only in one sample in this survey, but when infection is caused by intestinal bacteria, such as extended spectrum β-lactamases and carbapenem-resistant *Enterobacteriaceae,* the effect of antibacterial agents may not be acquired and a serious problem may occur.

MRSA was detected from SU covers, but not from SK covers, in this study. The survival rate of MRSA varies depending on the bacterial strain and various conditions, such as the material to which the bacterium is attached, temperature, and humidity 15-17. Notably, MRSA attached to covers may survive differently depending on the storage conditions. Therefore, SK covers contaminated with MRSA are predicted to be found if the number of samples is increased.

At our hospital, methods of handling and storing lightproof covers after human contact have not been examined previously. Based on this study's findings, we formulated the standards for the use of lightproof covers and enforced adherence to the disinfection manual. In the future, continuous examinations of and measures against bacterial contamination of lightproof covers, in cooperation with Infection Control Team, are required to determine the validity of the measures. Furthermore, pharmacists are expected to play additional roles in the management of steps preceding drug administration to patients in wards or at bedside. Moreover, development of lightproof covers made from new materials that prevent bacterial attachment to infusion bags and infusion preparations in bags is warranted.

## Figures and Tables

**Figure 1:**
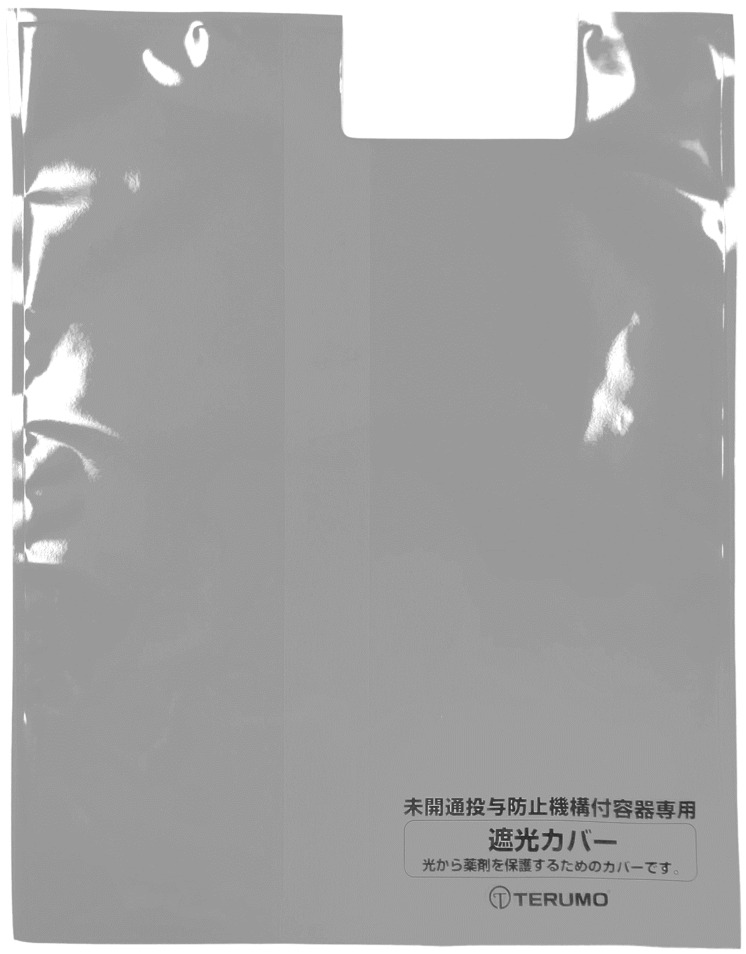
The lightproof covers.

**Table 1:** Bacterial culturing result of used lightproof covers in ward

No.	Ward	Outside	Inside	Period of use
SU1	5N	*Bacillus species*	(-)	Half day
SU2	5N	*Bacillus species*, CNS, MRSA	*Bacillus species*, CNS, MRSA	Half day
SU3	3S	*Bacillus species*, CNS, MRSA	*Bacillus species*, MRSA	1 day
SU4	3S	*Bacillus species*, CNS, MRSA	*Bacillus species*, CNS, MRSA	2 day
SU5	3S	*Bacillus species*, CNS, MRSA	*Bacillus species*, CNS, MRSA	4 day
SU6	3S	*Bacillus species*, CNS	*Bacillus species*, CNS	number of days unknown
SU7	3S	*Bacillus species*	CNS	number of days unknown
SU8	3S	*Bacillus species*, CNS	*Bacillus species*, CNS	number of days unknown
SU9	3S	*Bacillus species*, CNS	*Bacillus species*	number of days unknown

(-): no detection; CNS: coagulase-negative Staphylococci; MRSA: methicillin-resistant *Staphylococcus aureus;* 3S: medicine ward, pediatric ward, ophthalmology ward; 5N: medicine ward.

**Table 2:** Bacterial culturing results of lightproof covers stored in the ward

No.	Ward	Outside	Inside
SK1	5S	*Bacillus species*	*Bacillus species*
SK2	5S	*Bacillus species*, CNS	*Bacillus species*, Intestinal bacteria
SK3	5N	(-)	*Bacillus species*
SK4	5N	*Bacillus species*	*Bacillus species*
SK5	4S	CNS	CNS
SK6	4S	*Bacillus species*, CNS	CNS
SK7	3S	*Bacillus species*	*Bacillus species*
SK8	3S	*Bacillus species*	CNS

(-): no detection; CNS: coagulase-negative Staphylococci; 5S, 5N: medicine ward; 4S: surgical ward・urology ward, 3S: medicine ward, pediatric ward, ophthalmology ward.

**Table 3:** Bacterial culturing results of lightproof covers in the comparison group

	No.	Outside	Inside
Unused lightproof covers for pharmacy storage	CP1	(-)	(-)
	CP2	(-)	(-)
	CP3	(-)	(-)
Unused portion stored in the pharmacy department; lightproof covers installed in the ward for 24 hours	CW1	(-)	(-)
	CW2	(-)	(-)
	CW3	*Bacillus species*, CNS	(-)

(-): no detection; CNS: coagulase-negative Staphylococci.
